# Globally weaker and topologically different: resting-state connectivity in youth with autism

**DOI:** 10.1186/s13229-017-0156-6

**Published:** 2017-07-26

**Authors:** Benjamin E. Yerys, John D. Herrington, Theodore D. Satterthwaite, Lisa Guy, Robert T. Schultz, Danielle S. Bassett

**Affiliations:** 1 Center for Autism Research and Department of Child and Adolescent Psychiatry and Behavioral Sciences, Children’s Hospital of Philadelphia, 3535 Market Street, Ste 860, Philadelphia, PA 19104 USA; 2 Department of Psychiatry, Perelman School of Medicine, The University of Pennsylvania, Philadelphia, PA USA; 3 Department of Psychiatry, School of Medicine, University of North Carolina at Chapel Hill, Chapel Hill, NC USA; 4 Department of Pediatrics, The Children’s Hospital of Philadelphia, Philadelphia, PA USA; 5 Departments of Bioengineering and Electrical & Systems Engineering, University of Pennsylvania, Philadelphia, PA USA

**Keywords:** Attention, Autism spectrum disorder, Children, Intrinsic networks, Social cognition

## Abstract

**Background:**

There is a lack of agreement about functional connectivity differences in individuals with autism spectrum disorder (ASD). Studies using absolute strength have found reduced connectivity, while those using relative strength––a measure of system topology––reveal mostly enhanced connectivity. We hypothesized that mixed findings may be driven by the metric of functional connectivity.

**Methods:**

Resting-state echo planar 3 T functional magnetic resonance imaging scans were acquired on a Siemens Verio Scanner from 6 to 17-year-old youth with ASD (*n* = 81) and a matched typically developing control group (*n* = 82). All functional time series data were preprocessed using a confound regression procedure that has been previously validated in large-scale developmental datasets. It has also been shown to be highly effective at reducing the influence of motion artifact on connectivity data. We extracted time series data from a 333-node parcellation scheme, which was previously mapped to 13 functional systems. A Pearson’s correlation was calculated and transformed to Fisher’s *z* between every pair of nodes to create a weighted 333 × 333 adjacency matrix. Mean absolute functional connectivity strength was the mean Fisher’s *z* of the matrix. Relative functional connectivity was corrected for individual differences in mean absolute functional connectivity (i.e., each connection in the matrix was divided by their mean z), and functional connectivity was evaluated within and across each of the functional networks in the parcellation scheme.

**Results:**

Absolute functional connectivity strength was lower in ASD, and lower functional connectivity was correlated with greater ASD symptom severity. Relative functional connectivity was higher for the ASD group in the ventral attention and retrosplenial-temporal systems, with lower cross-system functional connectivity between the ventral attention and somatomotor-mouth systems. Functional connectivity within the ventral attention and retro-splenial systems correlated significantly with ASD symptom severity.

**Conclusions:**

Within a context of globally weaker functional connectivity, youth with ASD have an atypical topology of brain systems that support social perception and communication. This study clarifies the mixed results reported previously and demonstrates that the functional connectivity metric influences the observed direction of functional connectivity differences for individuals with ASD.

**Electronic supplementary material:**

The online version of this article (doi:10.1186/s13229-017-0156-6) contains supplementary material, which is available to authorized users.

## Background

Autism spectrum disorder (ASD) occurs in ~1.5% [1] of the population, a prevailing hypothesis is that perturbations of the functional connections among brain regions are associated with several functional impairments [2–5]. Evidence supporting this hypothesis consists of correlations between the functional time series of brain regions––termed functional connectivity––in youth and adults with ASD. Across the lifespan, individuals with ASD show differences in the strength of functional connectivity of brain regions that support a variety of social [2–12], cognitive [11–17], and sensorimotor functions [3, 11, 13, 16, 17]. Furthermore, these functional connectivity differences often correlate with ASD symptom severity [2–4, 18] and have been shown to correlate with adaptive behavior [5]. Despite the advances made in understanding functional connectivity perturbations that underlie core and associated features of ASD, significant questions remain regarding the brain’s functional organization (topology) in ASD.

A significant barrier in our understanding of brain organization in ASD is the disagreement on whether individuals with ASD demonstrate globally reduced or enhanced functional connectivity. Initial resting-state functional connectivity studies in ASD targeted regions comprising a single system^1^ (e.g., regions supporting vision) or a handful of systems [4–6, 9–12, 16, 17, 19–23]. These studies led to an early hypothesis of reduced functional connectivity in ASD [24]. However, this hypothesis has been challenged [3, 25, 26]. A limitation to this early research is that conclusions were being drawn from data that excluded multiple cognitive systems.

To date, three studies involving individuals with ASD and typically developing controls (TDCs) have examined functional connectivity using whole-brain parcellation schemes [3, 13, 27]. Two studies utilized functional network atlases that separated the brain into coarse large-scale systems (e.g., primary sensory, primary visual, subcortical), with mixed results regarding the overall pattern of differences for individuals with ASD. One of them observed increased functional connectivity in 13–15% of functional connections in youth with ASD compared to TDCs, and hyper-connectivity explained significant variance in social communication symptoms of ASD [3]. The other study observed primarily reduced functional connectivity in individuals with ASD across the lifespan and did not report correlations with ASD symptoms [13]. The third study which used a more detailed functional atlas reported poorer dissociation of networks for individuals with ASD via weaker cohesion within networks and greater dispersion across networks. This finding eschewed the traditional viewpoint of functional connectivity being globally enhanced or reduced in ASD [27]. These mixed findings leave gaps in our understanding of the topology of functional systems in ASD that need to be filled.

One key issue is purely methodological. Functional connectivity is usually characterized at both a global and a topological level across the whole brain. When looking for global functional connectivity properties, we evaluate the *absolute strength* of functional connections in the brain—do individuals with ASD have stronger or weaker connections among brain regions compared to those without ASD? When looking at the topology of functional connectivity patterns, we evaluate the *relative strength* of these connections—do individuals with ASD show a different pattern of functional connectivity within and across cognitive systems compared to those without ASD? While topology is often measured with graph-theory metrics in neuroscience [28], in the mathematics literature, the pattern of edge weights (i.e., functional connections between brain regions here) can be described as composing the network’s topology. The two studies that have examined whole-brain functional topology differed in their approach. The whole-brain study that reported reduced functional connectivity in ASD measured absolute strength of connections [13], whereas the other study that reported increased functional connectivity measured the *relative* strength of connections [3]. Thus, it is possible that youth with ASD may have globally weaker connections than typically developing youth and also demonstrate altered topology characterized by a greater relative strength in some systems compared to others. To our knowledge, no single study has examined overall absolute strength and relative strength of functional connections in the same study sample.

Another critical limitation of the aforementioned topological study in ASD is the general use of a coarse functional atlas. Coarse atlases combine multiple cognitive systems into one “association” or “hetermodal” system. The strength of this approach is that it leverages widely used brain atlases and thereby maximizes comparison to available evidence [3]; however, collapsing established functional systems (default mode, ventral attention, salience) into a global “association” system may blur important distinctions between systems and obscure their relationship to psychopathology. A growing literature points to the validity of using functional connectivity atlases that parse the brain into a number of cognitive systems. One such atlas––which parses the brain into 13 systems––provides a more homogeneous signal across voxels within each individual brain region than the atlases used in the studies above [29]. This atlas has been applied successfully in adults [30–33]. To our knowledge, this functional atlas has not been applied to the study of ASD.

The present study seeks to address these gaps in our understanding of whole-brain functional topology in youth with ASD. Based on the whole-brain studies of functional system topology in ASD [3, 13, 27], we predicted that measures of absolute strength would corroborate the previously reported global effect of reduced functional connectivity in youth with ASD [13]. Further, we predicted that measuring relative strength of systems would reveal enhanced functional connectivity in specific functional systems in youth with ASD [9, 11, 27]. Based on prior resting-state studies, we hypothesized that we would observe group differences in the default mode [7, 10, 12, 21, 22], ventral and dorsal attention [14], salience [8, 12], and somatomotor [16, 17] functional systems in youth with ASD. Finally, we investigated whether differences in both absolute strength and relative strength in specific systems would correlate with ASD symptom severity [3] or age [14].

## Methods

### Participants

A total of 214 youth (111 ASD and 103 TDC) between the ages of 6 and 17 participated in a resting-state functional MRI scan across multiple studies at the Center for Autism Research between 2010 and 2014. Youth in the ASD group met the *DSM-IV-TR* criteria for autism, asperger’s syndrome, or pervasive developmental disorder––not otherwise specified [34], informed by the Autism Diagnostic Interview––revised [35] and the Autism Diagnostic Observation Schedule (ADOS). We used the revised ADOS algorithm [36] that aligns with the second edition’s algorithm [37]. *DSM-IV-*TR criteria were used because data collection started prior to the release of the DSM-5, and we wanted to maintain diagnostic consistency in our sample across this group of studies. Youth with ASD were excluded if parents reported any known genetic, mood, psychotic, or neurological disorder, extreme premature birth (gestational age < 32 weeks), or other significant medical conditions that affected functioning. Thirty-nine youth were not prescribed medications at the time of the scan (48%), and seven of the 42 youth prescribed medications were prescribed more than one medication. Prescribed medications included stimulants (*n* = 21), selective serotonin reuptake inhibitors (*n* = 19), selective norepinephrine reuptake inhibitors (*n* = 5), alpha 2A agonists (*n* = 5), atypical antipsychotic (*n* = 3), and an aminoketone anti-depressant (*n* = 1). A subset of youth prescribed stimulant medication were asked to withhold their medication on the day of scanning, to minimize the effects of these medications on brain (*n* = 6). TDC participants were screened and excluded if parents reported any known genetic, language, learning, neurological, or psychiatric disorder, premature birth, any first- or second-degree relative with ASD, or receiving any psychoactive medication. TDC youth were also excluded if they presented with elevated symptoms on the parent reported Child and Adolescent Symptom Inventory [38]. We excluded 48 youth (ASD *n* = 28; TDC *n* = 20) with a mean framewise displacement >0.2 mm during fMRI scanning. Three more youth (ASD *n* = 2) were excluded because their global functional connectivity was >4 standard deviations from their own group’s mean. Thus, our final sample included 163 youth (ASD *n* = 81; TDC *n* = 82). The groups were matched on chronological age and sex-ratio, but not on General Conceptual Ability (GCA – analogous to full scale IQ) as measured by the Differential Ability Scales – Second Edition [39]; see Table 1 for group characteristics. Groups were well matched on in-scanner motion (mean root mean square displacement: ASD *M* = 0.10, SD = 0.04; TDC *M* = 0.11, SD = 0.04; *t*(158.79) = 1.13, *p* = 0.26, Cohen’s *d* = 0.25). As documented in post hoc analyses, we carried out a sensitivity analysis by matching our samples on cognitive ability (*p* = 0.90; An additional Table file shows this information (see Additional file 1)).

**Table 1:** Participant demographics and characterization

	ASD *n* = 81	TDC *n* = 82	*p* value
Age – M(SD)	149 ms (31 ms)	149 ms (33 ms)	0.97
GCA – M(SD)	106 (20)	112 (17)	*0.04*
Sex-ratio (M:F)	64:17	67:15	0.82
ADOS social affect	8.74 (3.59)	–	–
ADOS repetitive behaviors	2.33 (1.66)	–	–
ADOS total score	11.07 (3.81)	–	–
ADOS Calibrated Severity Score	6.44 (2.04)	–	–
ADHD-IV Rating Scale Total Score	24.10 (11.45)	4.28 (4.53)	*<.001*
CASI-IV – Anxiety (20 items)	12.49 (8.21)	1.84 (2.33)	*<.001*

*ADOS-2* Autism Diagnostic Observation Schedule, 2nd Edition, *ASD* autism spectrum disorder, *CASI* Child and Adolescent Symptom Inventory, 4th Edition, *GCA* General Conceptual Ability, *ms* months, *TDC* typically developing control

*P*-values in *italics* denote significant group differences

### Image acquisition

Functional images were acquired on a 3 T Siemens Verio scanner using a T2*-sensitive gradient echo pulse sequence: 160 whole-brain volumes, 40 slices, TR/TE/Flip angle/voxel size=2340/25 ms/60^o^/3.55 mm isotropic. Thirty-seven youth (20 ASD) received a slightly modified sequence: 172 whole-brain volumes, 36 slices, TR/TE/FOV/flip angle/voxel size = 2110/25 ms/60^o^/3.5 mm isotropic (with a .35 mm gap between slices). A high-resolution T1-weighted image for co-registration of the functional images was acquired with an MPRAGE sequence: TR/TE/voxel size/flip angle=300/2.46/1 mm isotropic/60^o^. Thirty-seven youth (20 ASD) received a slightly modified sequence: 172 whole-brain volumes, 36 slices, TR/TE/voxel size/flip angle=1900/2.54/0.8 × 0.8 × 0.9/9^°^. Participants were instructed to keep their eyes open and lie still while the monitor displayed a black screen.

### Subject-level time series processing

All functional time series data were preprocessed using a procedure that has been previously validated in large-scale developmental datasets and has been shown to be highly effective at reducing the influence of motion artifact on connectivity data [40–42]. A recent benchmarking paper comparing more than a dozen preprocessing pipelines for resting-state fMRI data demonstrated that the 36-parameter models are considered an optimal approach for pediatric group comparisons compared to the most common non-GSR pipeline (24-parameter) [43]. Preprocessing included removal of the first four volumes to allow for signal stabilization, slice time correction, realignment to the median volume, brain extraction, spatial smoothing (7 mm FWHM), and grand mean scaling. Mean white matter (WM) and cerebrospinal fluid (CSF) signals were extracted from the filtered time series data using tissue segments generated for each subject. Improved confound regression included nine standard confound signals (six motion parameters + global/WM/CSF) as well as the temporal derivative, quadratic term, and the temporal derivative of the quadratic term (36 parameters in total). We band-pass filtered the functional time series and the confound regressors simultaneously to retain frequencies between 0.01 and 0.08 Hz; identical temporal filtering prevented frequency mismatch between the confound parameters and the time series data [44].

### Image registration

The T1 image was skull stripped using FSL BET [45], bias corrected and segmented using multiplicative intrinsic component optimization [46], and registered to the Montreal Neurological Institute (MNI) template using a highly accurate deformable registration with attribute matching and mutual salience weighting [47]. Processed subject-level echo planar images were co-registered to the T1 image using boundary-based registration with integrated distortion correction as implemented in FSL5. All registrations were inspected manually.

All inferential statistics described below were calculated in the statistical programming language **R** [48]**.**

### Mean network strength analysis

**Fig. 1:**
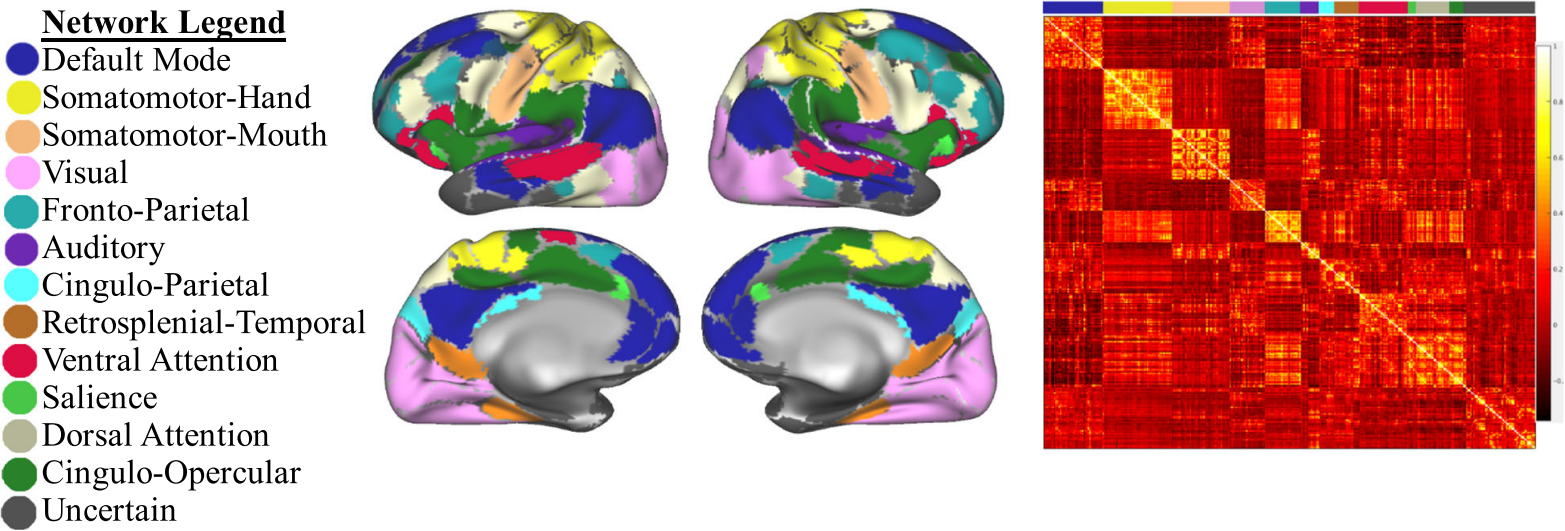
Functional brain network construction. We studied 333 regions of interest in cortical structures, but excluded the cerebellum. We calculated the pairwise correlation between time series as a measure of functional connectivity and encoded the results in a weighted adjacency matrix. In this figure, the adjacency matrix is the mean of the TDC group organized into the 13 cognitive systems previously defined in the literature (see color legend)

We extracted time series data from a 333-node parcellation scheme, which was previously mapped to 13 functional systems in an independent sample [29]. The 13 cognitive systems are visual, auditory, somatomotor hand (SMH), somatomotor mouth (SMM), default mode (DMN), fronto-parietal (FP), cingulo-opercular (CO), cingulo-parietal (CP), salience, dorsal attention (DA), ventral attention (VA), and retrosplenial-temporal (RT). Parcels not assigned to a system (“Uncertain”) were not evaluated. We then estimated functional connectivity between every pair of nodes to create a weighted 333 × 333 adjacency matrix, which represents each participant’s functional brain network (see Fig. 1). We calculated the mean absolute functional connectivity strength for each participant by transforming each correlation in the 333 × 333 adjacency matrix using a Fisher’s *z* statistic and then calculating the mean of the matrix. This global measure was compared between ASD and TDC groups with a stepwise linear regression while accounting for major known confounding variables, including age, sex, IQ, in-scanner motion (i.e., relative mean displacement), and scan sequence. We report both effect sizes and the 95% confidence interval (*CI*) in addition to *F* tests and *p* values. We examined the relationship between a potential main effect in mean strength and ASD symptom severity from the ADOS using both raw scores and the Calibrated Severity Scores. Spearman’s *rho* was used for this analysis as scores from the ADOS do not have equal intervals and a normal distribution [49], and Pearson correlations were used for age.

### Analysis of the topological architecture of functional networks

To examine system topology, we calculated within- and cross-system connectivity from the same weighted adjacency matrix as above. Within-system connectivity is the mean strength of the functional interactions within a system (e.g., mean of all connections among DMN nodes). Cross-system connectivity of two systems is the mean strength of functional interactions between ROIs of one system (e.g., DMN) and ROIs of another system (e.g., Salience). Here, the Fisher’s *z* adjacency matrix is divided by each individual’s global mean Fisher’s *z* to maximize sensitivity to topological structure and account for individual differences in mean connectivity strength (see additional figures for distribution of mean connectivity strength pre- and post-normalization [Additional file 2]). This approach has been used in a large-scale study of typical development [32], and in a prior study of whole-brain topology in ASD [3]. Normalized correlations are ideally suited for interrogating the topological architecture of functional systems because they control for individual differences in the total weight of connections.

We compared the within-system and cross-system functional connectivity of all normalized systems for ASD and TDC groups while accounting for the major confounding variables of age, IQ, sex, and scan sequence. All within-system and cross-system differences were corrected with a False Discovery Rate (FDR; *q* < 0.05) on the effect of group in the ANCOVA model while accounting for the number of comparisons for each type of analysis (i.e., 12 for within-system and 66 for cross-system). For each within-system (or cross-system) connection that differed between groups, we examined the relationship with ASD symptoms using raw and calibrated severity scores from the ADOS.

### Post hoc analyses

Due to the ongoing controversy of including global signal regression in our 36 parameter models, we re-evaluated our significant group differences with a 24 parameter models that excluded covariates related to global, white matter, and cerebro-spinal fluid signal. While we included MRI sequence as a covariate in our original analysis, we also evaluated whether there were significant interactions between group effects and MRI sequence. We conducted a sensitivity analysis to examine how our findings may (or may not) change with better group matching of IQ rather than entering it as a covariate in all analyses. To accomplish this goal, we individually matched children within 1 standard deviation on GCA and removed all participants with ASD that had a GCA score below the lowest TDC score. We also evaluated the global participation coefficient between groups. This measure quantifies how connected a node is to other nodes within and between systems [50] instead of evaluating a grand functional connectivity mean. Thus, this complementary measure of global functional connectivity indexes the number of connections across nodes instead of overall strength. Finally, we explored the relationship of age to our key topology findings.

## Results

### Diminished absolute mean connectivity strength in youth with ASD

**Fig. 2:**
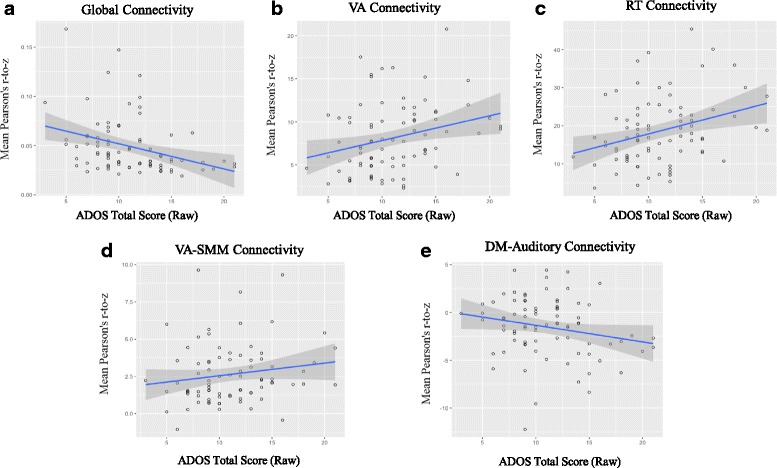
Relationship between network statistics and symptomatology. Here, we show the relationship between the total score on the ADOS (revised algorithm) and **a** global connectivity, and **b** connectivity within VA=ventral attention, **c** connectivity within RT=retrosplenial-temporal, **d** connectivity between VA-SMM=ventral attention to somatomotor-mouth, **e** connectivity between DM-auditory=default mode to auditory

The absolute mean connectivity strength was lower in youth with ASD (Fisher’s *z M* = 0.05, SD = 0.03) than TDC (Fisher’s *z M* = 0.07, SD = 0.05) after controlling for age, sex, IQ, in-scanner motion, and scan sequence, *F*(1, 156) = 6.50, *p* = 0.01, *η* ^*2*^_*p*_ = 0.04, 95% *CI* = [−0.007, −0.032]). Furthermore, within the ASD group, there was a significant negative correlation between mean connectivity strength and the overall score on the ADOS (raw: *ρ*(81) = −0.39, *p* < 0.001; calibrated severity: *ρ*(81) = −0.35, *p* = 0.013; see Fig. 2). These results demonstrated that youth with ASD had significantly weaker global functional connectivity and that those with the weakest global functional connectivity had the most severe ASD symptoms.

### Topology of functional systems

**Fig. 3:**
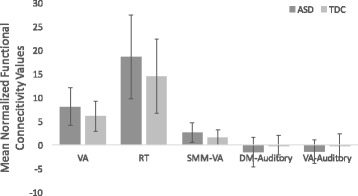
Group differences in network architecture. Here, we show group differences of relative strength for within and cross-system functional connectivity. Error bars represent the standard deviation

These analyses revealed *higher* within-system functional connectivity for the ASD group compared to the TDC group with an uncorrected *p* < 0.05 for the VA, RT, DM, SMH, and SMM systems indicating that the topology of system connectivity strengths differed for ASD and TDC groups. However, only the VA (*F*(1, 156) = 9.00, FDR-corrected *p* = 0.004, *η* ^*2*^_*p*_ = 0.05, 95% *CI* = [0.88, 3.11]) and the RT system (*F*(1156) = 7.20, FDR-corrected *p* = 0.048, *η* ^*2*^_*p*_ = 0.04, 95% *CI* = [1.51, 6.69]) survived multiple comparisons correction. Cross-system analyses revealed *lower* functional connectivity for the ASD group between the VA-SMM systems (*F*(1156) = 11.27, FDR-corrected *p* = 0.044, *η* ^*2*^_*p*_ = 0.07, 95% *CI* = [−0.46, −1.58]), the DM-Auditory systems (*F*(1156) = 10.67, FDR-corrected *p* = 0.044, *η* ^*2*^_*p*_ = 0.06, 95% *CI* = [−0.70, −2.36]), and a marginal finding in the VA-Auditory systems (*F*(1156) = 9.51, FDR-corrected *p* = 0.053, *η* ^*2*^_*p*_ = 0.06, 95% *CI* = [−0.63, −2.14]). See Fig. 3 and additional table files for within- and cross-system means [Additional files 3 and 4].

### Attention and social cognition system abnormality correlates with ASD symptoms

Within the ASD group, the overall score on the ADOS was positively correlated with the VA’s within-system functional connectivity (raw: *ρ*(*n* = 81) = 0.29, FDR-corrected *p* = 0.022; calibrated severity: *ρ*(*n* = 81) = 0.24, FDR-corrected *p* = 0.071; see Fig. 2b), and the RT’s within-system functional connectivity (raw: *ρ*(81) = 0.30, FDR-corrected *p* = 0.023; calibrated severity: *ρ*(*n* = 81) = 0.26, FDR-corrected *p* = 0.071). The VA-SMM cross-systems functional connectivity was marginally correlated with the ADOS (raw: *ρ*(*n* = 81) = 0.20, FDR-corrected *p* = 0.101; calibrated severity: *ρ*(*n* = 81) = 0.16, FDR-corrected *p* = 0.187). The ADOS scores were negatively correlated with functional connectivity of the DM-auditory systems at a marginal level of significance (raw: *ρ*(81) = −0.22, FDR-corrected *p* = 0.078; calibrated severity: *ρ*(*n* = 81) = −0.21, FDR-corrected *p* = 0.101). The correlation between the ADOS and the VA-auditory system was weak and not significant (raw: *ρ*(81) = −0.07, uncorrected *p* = 0.527; calibrated severity: *ρ*(*n* = 81) = −.05, FDR-corrected *p* = 0.676).

### Results of post hoc analyses

We examined if our results of group differences were influenced by the use of two sequences by probing for group by sequence interactions. For absolute strength connectivity, the interaction was non-significant, sequence 1 mean (SD) = 0.061 (0.04); sequence 2 mean = 0.052 (0.03), *F*(1155) = 1.15, *p* = 0.29, *η* ^*2*^_*p*_ *=* 0.01, 95% CI [−.04, .01], as was the case for all relative strength functional connectivity differences (FDR-corrected *p*’s > 0.72, *η* ^*2*^_*p*_ < 0.02).

We created a subset of 60 participants from each group well-matched on age, IQ, and sex ratio (all *p*’s ≥ 0.80, Cohen’s *d* ≤ 0.05). Group differences in overall functional connectivity strength and functional connectivity within the VA and VA-SMM remained significant in this subset with a trend toward replicating the difference in the RT system. Correlations with symptoms were also replicated in the subset for overall strength, RT and VA systems (see Additional files 5 and 6 for detailed results). Another robustness analysis used the mean participation coefficient across the 333 nodes as a global measure of functional connectivity; the main effect of group was significant (*F*(1, 156) = 4.81, *p* = 0.03; *η* ^*2*^_*p*_ = 0.03, 95% CI = [−0.001, −0.012]). The ASD group (*M* = 0.82, SD = 0.02) had a lower participation coefficient than the TDC group (*M* = 0.83, SD = 0.02). Age was significantly and negatively correlated with the functional connectivity within the VA (*r*(81) = −0.25, *p* = 0.03) as well as significantly and negatively correlated with the functional connectivity between the VA-SMM systems (*r*(81) = −0.32, *p* = 0.004. All other correlations with age were small effects and non-significant (*r*’s ≤ 0.15; *p*’s > 0.17).

## Discussion

We applied a whole-brain approach to identify both global and topological functional connectivity differences in youth with ASD. As predicted, youth with ASD demonstrated overall reduced absolute functional connectivity strength relative to TDCs. Topological analyses showed that the ASD group had *higher* relative functional connectivity strength within the attention and social cognition systems compared to the TDC group and diminished connectivity between these and sensory systems. Furthermore, the connectivity metrics for global and topological analyses showed relationships between functional connectivity and ASD symptoms; though not all topological metrics correlated with ASD symptoms. Post hoc analyses evaluating MRI sequence effects and sensitivity analyses confirmed the main results. These findings support a novel conceptualization of brain organization in ASD: in the context of globally weaker functional connections, systems supporting attention and social cognition are more segregated (higher within-system and lower cross-system connectivity). This is important because it establishes a new framework for understanding the connectomics of ASD––a framework where systems’ absolute functional connectivity strength is considered separately from the topography of systems. Furthermore, these results clarify the mixed findings in the literature on functional connectivity in schoolage youth with ASD.

When using a measure of absolute functional connection strength, reduced connectivity has been found nearly all of the time in whole-brain and system-specific studies in individuals with ASD across multiple labs and samples of varying ages and cognitive abilities [4, 9–11, 13, 14, 16, 19–21] but see [3, 23, 51]. Many of these studies have also linked reduced functional connectivity to ASD symptom severity [4, 10, 11, 16, 19–21]. Our analysis of absolute functional connectivity strength replicated both this group difference and the relationship with ASD symptom severity. These are important findings that help to resolve ambiguities in the literature. Indeed, recent studies have shown both hypo- and hyper-connectivity observed in the same systems (e.g., DM) across labs with similar populations, similar handling of early preprocessing pipelines for dealing with motion, but different approaches to measuring functional connectivity strength [3, 6, 13, 19, 21, 22, 51]. Many papers, including our own [21], have attempted to resolve the mixed findings by attributing them to either subtle differences in participants’ age and functioning level or to the reliance on small and variable samples. While these factors may play a role, the present study shows that the distinction between absolute and relative functional connectivity is critical when interpreting inconsistent findings in ASD to date. Furthermore, our sensitivity analysis demonstrates that IQ differences between ASD and TDC groups could not explain our findings. Thus, the present study provides strong evidence that overall functional connectivity strength is reduced in youth with ASD and that it is associated with individual differences in symptom severity.

When examining relative functional connection strength, the ASD group showed enhanced functional connectivity within the VA system relative to the TDC group, but decreased functional connectivity between the VA and SMM systems. These differences were observed in the full sample and in sensitivity analyses. These systems are known to be atypical in youth with ASD. The VA system is responsible for responding to exogenous visual information. Prior research has found increased functional connectivity strength within the VA system in youth with ASD [14] and atypical activation to meaningful stimuli during the “alerting” phase of an attention task [52]. The SMM system is responsible for processing incoming sensory input and projecting motor output to the mouth and tongue [29], this motor output is critical for verbal and nonverbal communication. Previous research has demonstrated altered functional connectivity in the SMM system during rest in youth with ASD, and these perturbations have correlated with both more impaired motor skills and more severe social skills [53–55].

Relative strength analyses also revealed increased functional connectivity within the RT system in ASD. RT is often considered part of the DM system and involved in social cognition [56]. The enhanced functional connectivity in the RT systems converges with a prior study in youth with ASD where the ASD group’s retrosplenial cortex had increased absolute functional connectivity with multiple temporal lobe regions [22]. Neither study demonstrated that this atypical RT connectivity correlated with ASD symptom severity.

Evaluation of relative strength for cross-system functional connectivity revealed decreased connectivity between the VA-auditory and DM-auditory systems for youth with ASD compared to controls. These cross-system differences were observed in the full sample, but did not remain significant in our sensitivity analyses where we matched groups more rigorously on IQ. This suggests the findings may not be a reliable difference specific to ASD and that the extended IQ range in the ASD group may have driven them.

This study is the first to integrate absolute and relative functional connection strength in the same sample. Evaluating both forms of connection strength simultaneously provides an important step in advancing our knowledge of brain organization in ASD. This study reveals that the brain's functional connections are weaker overall in ASD, and this is associated with a compensation of greater segregation for attention, social cognition, and somatomotor systems. These system-specific findings align with imaging studies of toddlers [57] and younger siblings at risk for ASD [58, 59] that demonstrate unusual growth of white matter tracts linking attention and motor systems. Thus, one could speculate that early white matter development may influence the weighting of functional systems across development.

This pattern of altered system dynamics (i.e., greater segregation of systems and poorer integration between systems) can help bring clarity to seemingly contradictory findings on the topology of brain systems in ASD. One study used a relative strength measure of functional connectivity in youth with ASD and reported enhanced functional connectivity for a subset of connections in youth with ASD [3]—the present study partially replicates this finding by showing that youth with ASD have enhanced functional connectivity for within-system functional connectivity, but not for cross-system functional connectivity. The prior and present studies differed methodologically in ways that could affect the cross-system results. One notable difference is that Supekar and colleagues [3] sampled youth between 7 and 13 years of age, whereas the present study sampled a broader age range of 6–17 years, suggesting possible differences in findings due to the inclusion of older adolescents in the present study. The negative correlation between age and VA-SMM functional connectivity supports this argument as the oldest participants with ASD in the present study demonstrated the weakest functional connectivity. This same negative relationship with age was also found for the VA’s within-system functional connectivity; this finding also converges with a developmental study on the VA system in ASD [14]. Another methodological difference in the present study included the use of global signal regression to control for head motion in this study. There is an active discussion on best practices for reducing head motion, but current evidence from independent research groups suggests that global signal regression is an effective method [60, 61].

The other studies of topology have shown the opposite pattern from the present study; that is, prior studies showed that individuals with ASD demonstrated poor segregation and greater integration of systems in a limited set of systems [2, 9, 11] and a whole-brain analysis like the present one [27]. The present study differs from prior studies by controling for individual differences in functional connectivity strength with a relative functional connectivity measure that normalized individual connections by the mean of absolute functional connectivity strength across all connections. Prior studies used a binary measure of *r*’s > 0 included as a functional connection [27], or a measure of absolute strength [2, 9], or by taking an equivalent percentage of the strongest connections (i.e., sparsity approach) [11]. Thus, our study likely conflicts with these prior studies because the denominator of our normalized correlation is the mean absolute functional connectivity strength, which is lower in ASD. This led to the larger relative strength scores for the ASD group. The advantages of the present study’s approach to topology is that it controlled for individual differences in functional connectivity strength (uncontrolled with absolute strength approaches) while maintaining a weighted functional connection instead of a binary one, and evaluated all possible connections within our matrix instead of a limited number (i.e., sparsity approach).

This study is limited by use of a convenience sample that under-represents those with ASD and significant intellectual disability. While this limitation is common among resting-state functional connectivity studies, it does affect generalizability to those with intellectual disability. Nevertheless, this study is the largest ASD sample reported from a *single site and scanner*, to our knowledge. Thus, we avoided the pitfalls of small sample studies [62], while maintaining diagnostic fidelity across clinicians using a standardized diagnostic battery. This large sample afforded the study adequate power to reliably detect medium-sized effects in group differences and correlations with ASD symptoms. It should be noted that our use of FDR for multiple comparisons correction is more stringent than other approaches, such as the network based statistic [63], and therefore, other analytic approaches may have yielded more group differences. However, the findings reported here represent group differences that were robust to a number of important confounds addressed in our post hoc analyses. Finally, correlations between functional connectivity and the calibrated severity score were weaker than correlations with raw scores from the ADOS. While the calibrated severity scores are helpful for comparing symptom severity across modules, it restricts the ranges of scores to a 10-point scale that does not exist in the raw scores. Thus, we place more emphasis on the correlations between functional connectivity and raw score correlations because they allow for a broader range of symptom severity in our sample.

## Conclusions

In conclusion, this study highlights a nuanced view of functional connectivity in ASD, such that within the context of generally diminished functional connectivity, the topology of attention, social cognition, and somatomotor systems is enhanced [64]. Furthermore, the present study highlights that the specific metric of absolute vs. relative strength likely plays a role in many of the conflicting results observed previously. Thus, future attempts to summarize findings across studies or formal meta-analyses should note the dependent variable metric.

## Additional files

Additional file 1: IQ matched subset. **Table S1**. Participant characteristics for the matched subset within 12 months of age, 1 SD in IQ, and gender (when possible). (DOCX 55 kb)

Additional file 2: Connectivity distribution. **Figures S1–S24**. Distribution of raw and normalized correlations for 12 systems of interest. (DOCX 19579 kb)

Additional file 3: Within system connectivity. **Table S2**. Means of within-system functional connectivity for normalized correlations by group. (DOCX 58 kb)

Additional file 4: Cross-system connectivity. **Table S3**. Means of cross-system functional connectivity for normalized correlations by group (DOCX 94 kb)

Additional file 5: Follow-up groupdiff IQ matched. Means of cross-system functional connectivity for normalized correlations by group. (DOCX 65 kb)

Additional file 6: Follow-up correl IQ matched. Means of cross-system functional connectivity for normalized correlations by group. (DOCX 41 kb)

## Abbreviations

ADOS: Autism Diagnostic Observation Schedule
ASD: Autism spectrum disorder
*CI*: Confidence interval
CO: Cingulo-opercular
CP: Cingulo-parietal
CSF: Cerebrospinal fluid
DA: Dorsal attention
DMN: Default mode
*DSM-IV-TR*: Diagnostic and Statistical Manual of Mental Disorders, 4th Edition, text revision
FDR: False discovery rate
FP: Fronto-parietal
FWHM: Full width at half maximum
GCA: General Conceptual Ability
MNI: Montreal Neurological Institute
RT: Retrosplenial-temporal
SMH: Somatomotor hand
SMM: Somatomotor mouth
TDC: Typically developing control
VA: Ventral attention
WM: White matter

## Footnotes

1 In this paper, we take a network theory approach where the functional connectivity of the human brain is considered a network, and subcomponents are referred to as “systems”.
