# Supplementary material for: Globally weaker and topologically different: resting-state connectivity in youth with autism
Source: Mol Autism. 2017 Jul 26;8:39. doi: 10.1186/s13229-017-0156-6 (PMC5530457; doi:10.1186/s13229-017-0156-6)
Supplement: Supplementary file 1 — IQ matched subset. Table S1. Participant characteristics for the matched subset within 12 months of age, 1 SD in IQ, and gender (when possible). (DOCX 55 kb) [file 13229_2017_156_MOESM1_ESM.docx]

Table S1. Participant characteristics for the matched subset within 12 months of age, 1 SD in IQ, and gender (when possible).

|  | ASD  n=60 | TDC  n=60 | *p*-value |
| --- | --- | --- | --- |
| Age – M(SD) | 148 ms (32ms) | 149 ms (33ms) | 0.80 |
| GCA – M(SD) | 110 (18) | 110 (17) | 0.90 |
| Sex-Ratio (M:F) | 48:12 | 49:11 | 1.00 |
| ADOS-2 Social Affect | 8.97 (3.57) | -- | -- |
| ADOS-2 Repetitive Behaviors | 2.23 (1.60) | -- | -- |
| ADOS-2 Total Score | 11.20 (3.59) | -- | -- |

ADOS-2=Autism Diagnostic Observation Schedule, 2^nd^ Edition; ASD=Autism Spectrum Disorder; CASI=Child and Adolescent Symptom Inventory, 4^th^ Edition; GCA=General Conceptual Ability; ms=months; TDC=Typically Developing Control
