# Supplementary material for: Globally weaker and topologically different: resting-state connectivity in youth with autism
Source: Mol Autism. 2017 Jul 26;8:39. doi: 10.1186/s13229-017-0156-6 (PMC5530457; doi:10.1186/s13229-017-0156-6)

Distribution of the mean within-system connectivity of individual systems by group

Figure S1. A histogram of raw correlations for the default mode system by group


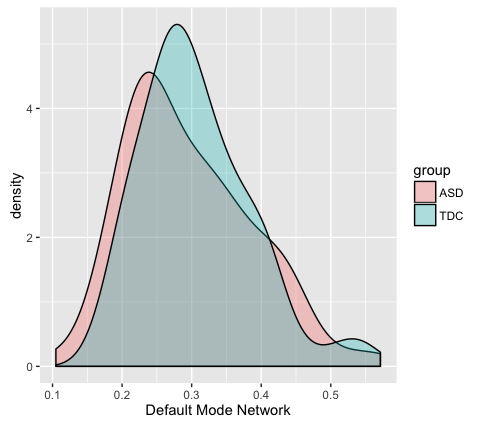


Figure S2. A histogram of normalized correlations for the default mode system by group


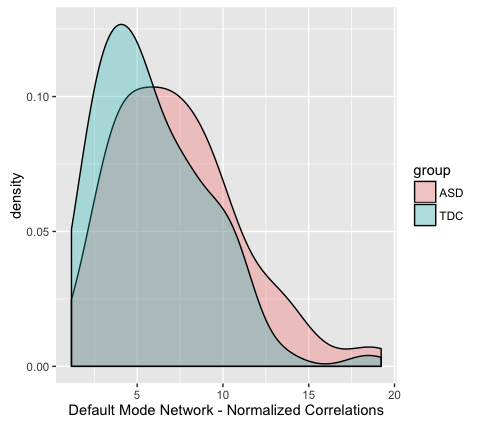


Figure S3. A histogram of raw correlations for the ventral somatomotor (hand) system by group


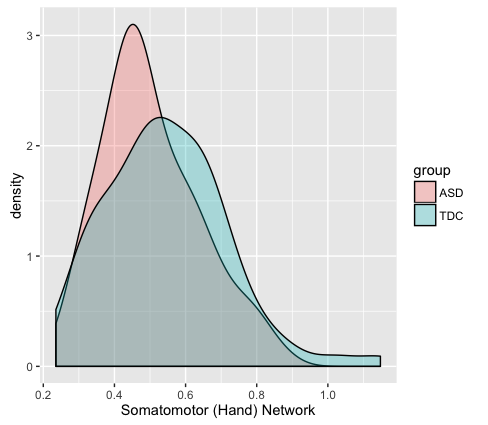


Figure S4. A histogram of normalized correlations for the somatomotor (hand) system by group


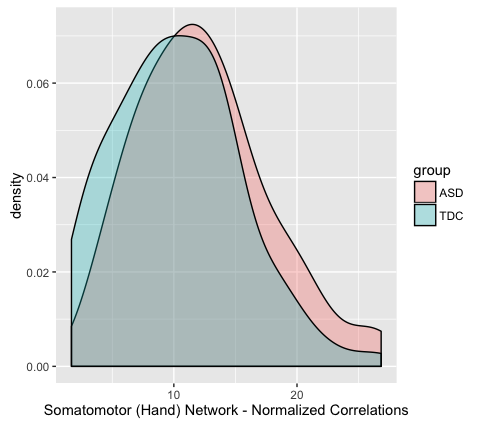


Figure S5. A histogram of raw correlations for the somatomotor (mouth) system by group


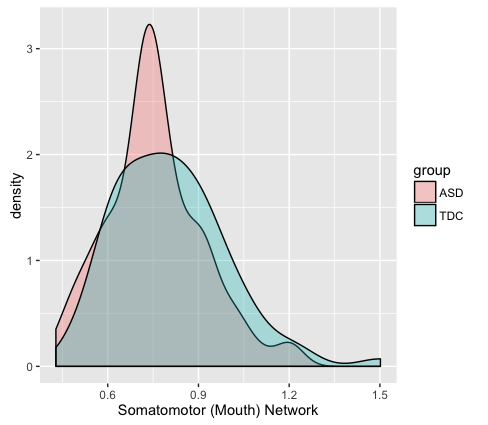


Figure S6. A histogram of normalized correlations for the somatomotor (mouth) system by group


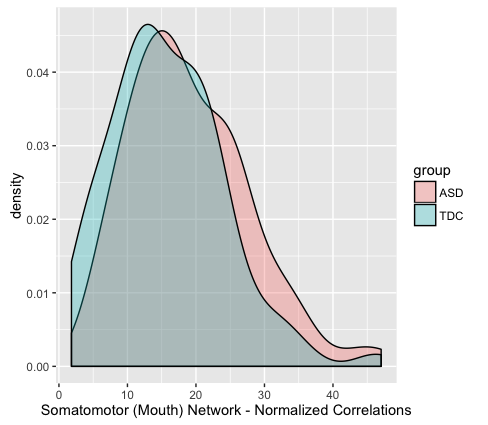


Figure S7. A histogram of raw correlations for the visual system by group


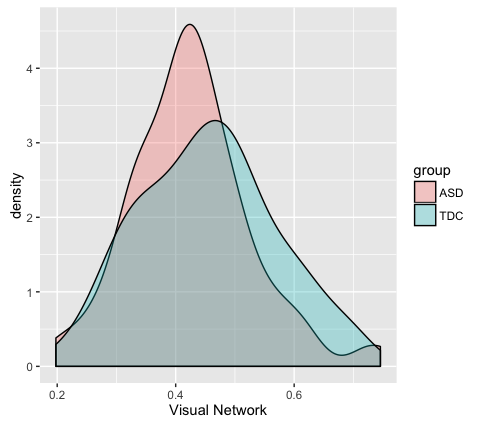


Figure S8. A histogram of normalized correlations for the visual system by group


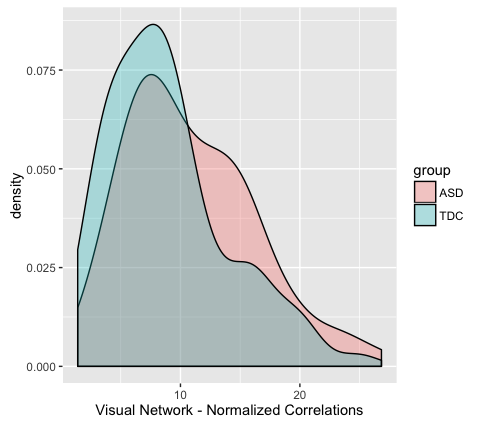


Figure S9. A histogram of raw correlations for the fronto-parietal system by group


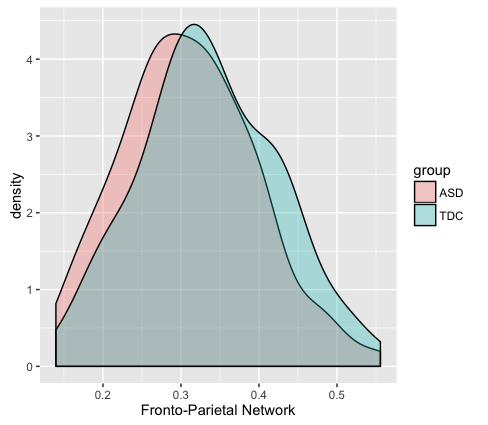


Figure S10. A histogram of normalized correlations for the fronto-parietal system by group


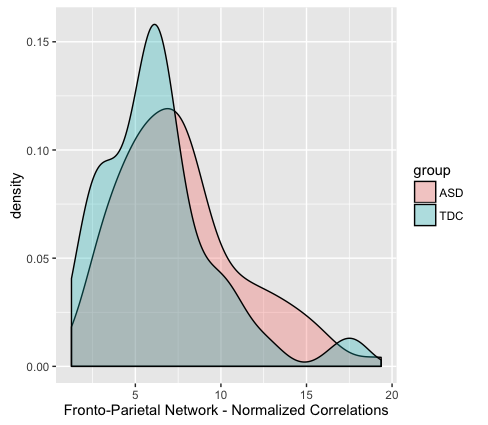


Figure S11. A histogram of raw correlations for the cingulo-opercular system by group


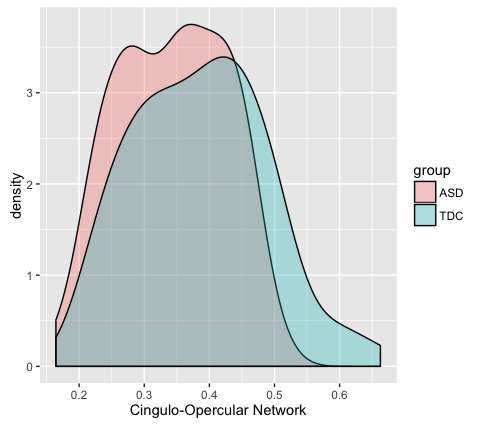


Figure S12. A histogram of normalized correlations for the cingulo-opercular system by group


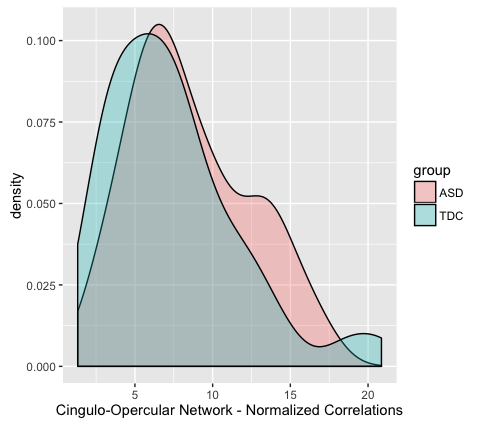


Figure S13. A histogram of raw correlations for the cingulo-parietal system by group


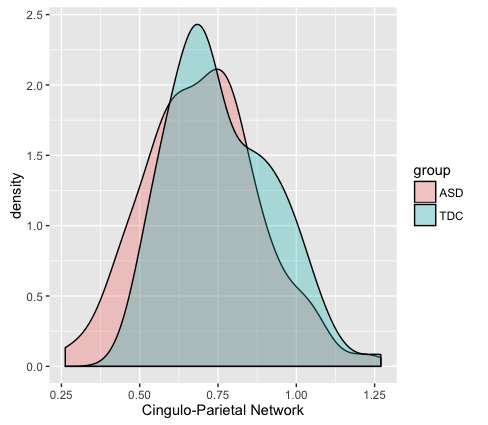


Figure S14. A histogram of normalized correlations for the cingulo-parietal system by group


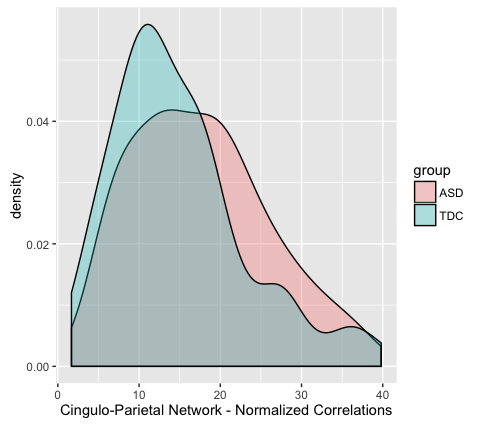


Figure S15. A histogram of raw correlations for the retrosplenial-temporal system by group


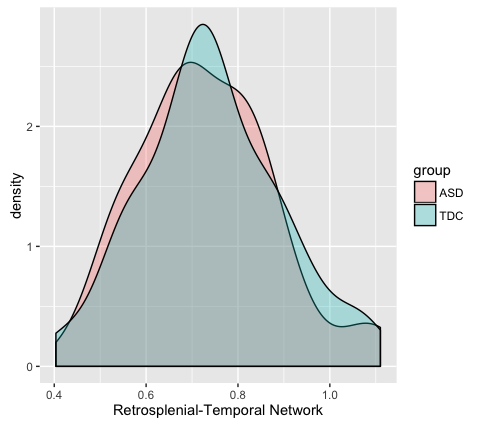


Figure S16. A histogram of normalized correlations for the retrosplenial-temporal system by group


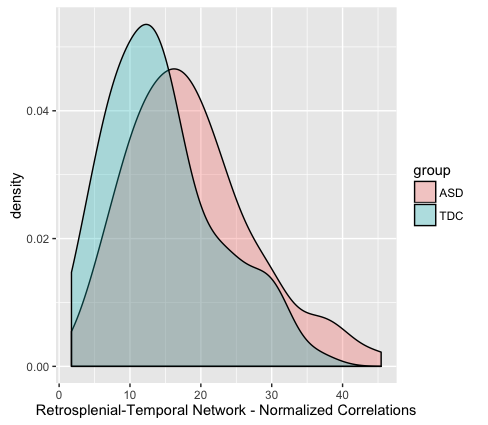


Figure S17. A histogram of raw correlations for the auditory system by group


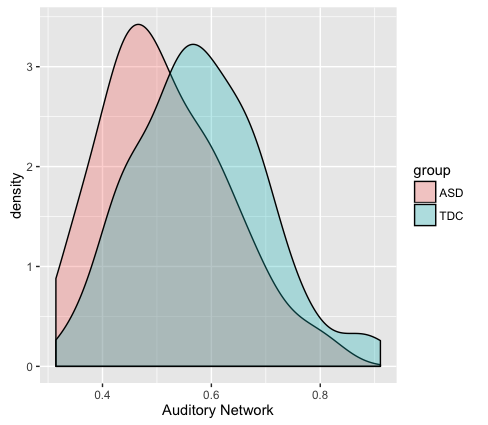


Figure S18. A histogram of normalized correlations for the auditory system by group


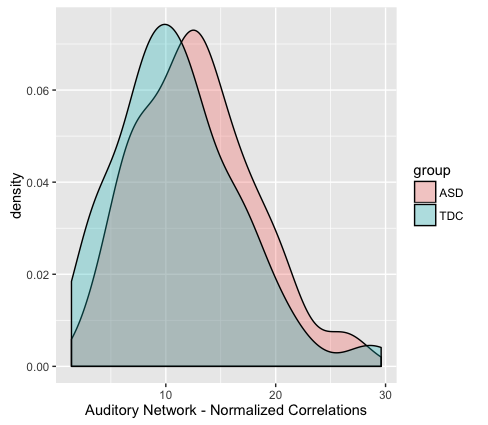


Figure S19. A histogram of raw correlations for the salience system by group


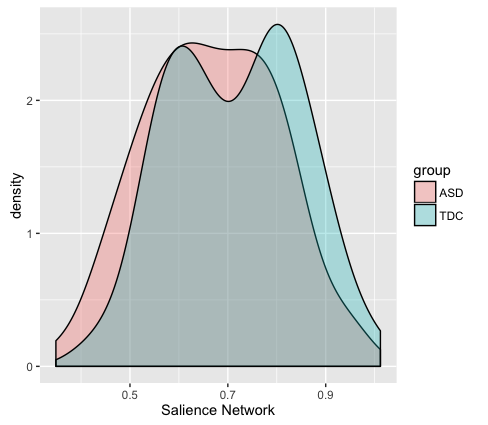


Figure S20. A histogram of normalized correlations for the salience system by group


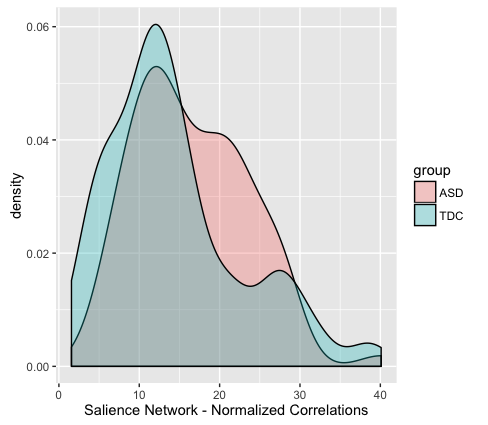


Figure S21. A histogram of raw correlations for the ventral attention system by group


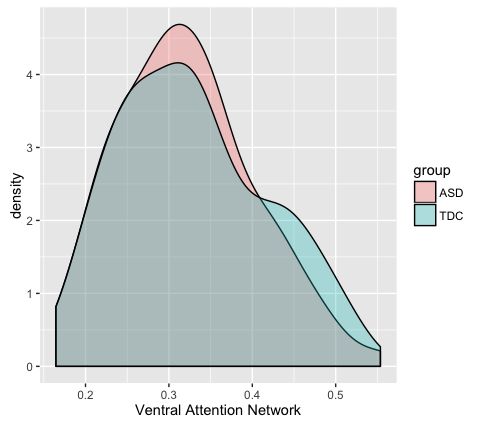


Figure S22. A histogram of normalized correlations for the ventral attention system by group


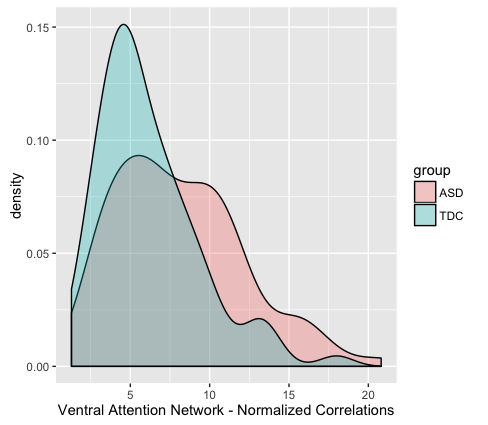


Figure S23. A histogram of raw correlations for the dorsal attention system by group


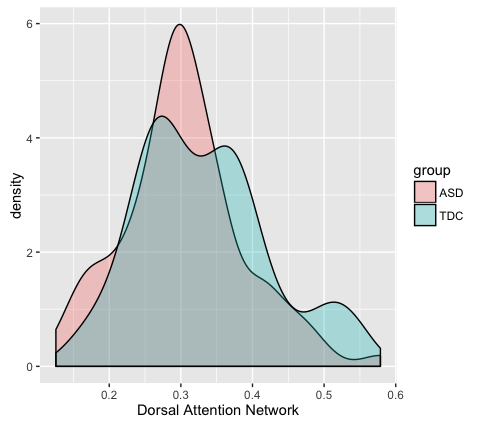


Figure S24. A histogram of normalized correlations for the dorsal attention system by group


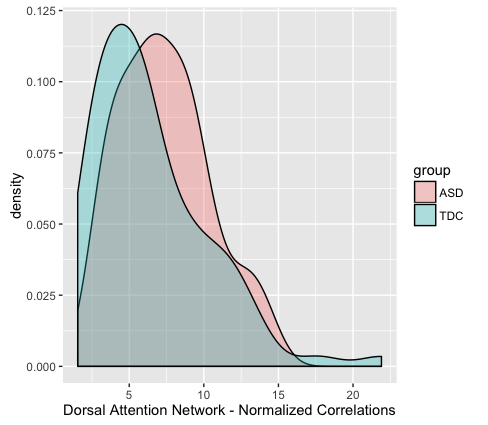

Supplement: Supplementary file 2 — Connectivity distribution. Figures S1–S24. Distribution of raw and normalized correlations for 12 systems of interest. (DOCX 19579 kb) [file 13229_2017_156_MOESM2_ESM.docx]
