# Supplementary material for: Globally weaker and topologically different: resting-state connectivity in youth with autism
Source: Mol Autism. 2017 Jul 26;8:39. doi: 10.1186/s13229-017-0156-6 (PMC5530457; doi:10.1186/s13229-017-0156-6)
Supplement: Supplementary file 3 — Within system connectivity. Table S2. Means of within-system functional connectivity for normalized correlations by group. (DOCX 58 kb) [file 13229_2017_156_MOESM3_ESM.docx]

Table S2. Means of within-system functional connectivity for normalized correlations by group $\frac{Fisher^{'}s z of each functional connection}{Global Functional Connecitivty Fisher^{'}s z}$

| Networks | ASD  *M*(SD) | TDC  *M*(SD) |
| --- | --- | --- |
| VA | 8.14 (4.00) | 6.15 (3.16) |
| RT | 19.19 (9.52) | 14.87 (8.40) |
| DM | 7.42 (3.69) | 5.94 (3.24) |
| SMH | 12.92 (6.09) | 10.43 (5.35) |
| SMM | 19.08 (9.20) | 15.33 (8.49) |
| Visual | 11.20 (5.90) | 9.10 (5.45) |
| Auditory | 13.63 (6.08) | 11.71 (6.30) |
| FP | 8.43 (4.34) | 6.93 (4.04) |
| DA | 8.03 (3.56) | 6.99 (4.43) |
| CO | 9.36 (4.57) | 7.99 (4.81) |
| CP | 18.09 (9.09) | 15.05 (8.62) |
| SAL | 18.33 (8.54) | 15.53 (9.43) |

CO=Cingular-Opercular

CP=Cingulo-Parietal

DM= Default Mode

FP=Frontoparietal

RT= Retrosplenial-Temporal

SAL=Salience

SMH= Somatomotor – Hand

SMM= Somatomotor – Mouth

VA= Ventral Attention
