# Supplementary material for: Globally weaker and topologically different: resting-state connectivity in youth with autism
Source: Mol Autism. 2017 Jul 26;8:39. doi: 10.1186/s13229-017-0156-6 (PMC5530457; doi:10.1186/s13229-017-0156-6)
Supplement: Supplementary file 4 — Cross-system connectivity. Table S3. Means of cross-system functional connectivity for normalized correlations by group (DOCX 94 kb) [file 13229_2017_156_MOESM4_ESM.docx]

Table S3. Means of cross-system functional connectivity for normalized correlations

| Systems | ASD  *M*(SD) | TDC  *M*(SD) |
| --- | --- | --- |
| **SMM-VA** | **2.64(2.01)** | **1.62(1.58)** |
| **DM-AUD** | **-1.53(3.15)** | **-0.01(2.10)** |
| **AUD-VA** | **-1.40(2.46)** | **-0.01(2.42)** |
| DM-SMH | -1.96(2.18) | -1.32(2.04) |
| DM-SMM | -2.13(2.39) | -1.39(2.51) |
| DM-VIS | 6.90(4.80) | 6.26(4.80) |
| DM-FP | -2.45(2.53) | -2.02(2.09) |
| DM-CP | 0.35(2.69) | 0.36(2.00) |
| DM-RT | 2.31(1.77) | 2.01(1.44) |
| DM-VA | -1.81(2.51) | -1.79(2.37) |
| DM-SAL | -2.23(2.74) | -1.97(2.70) |
| DM-DA | -2.25(2.17) | -2.07(2.17) |
| DM-CO | -1.42(2.29) | -1.24(1.89) |
| SMH-SMM | 6.38(4.17) | 6.54(3.98) |
| SMH-VIS | -1.01(2.80) | 0.03(2.33) |
| SMH-FP | -2.40(2.46) | -2.03(2.18) |
| SMH-AUD | 1.51(2.82) | 1.60(2.00) |
| SMH-CP | -2.48(3.35) | -2.48(3.35) |
| SMH-RT | -1.55(3.73) | -2.33(3.69) |
| SMH-VA | 2.41(3.07) | 2.35(2.74) |
| SMH-SAL | 1.73(2.60) | 1.48(2.14) |
| SMH-DA | -1.08(2.65) | -1.69(2.60) |
| SMH-CO | 1.28(2.43) | 1.00(1.96) |
| SMM-VIS | -0.61(3.97) | -0.66(2.65) |
| SMM-FP | 6.51(3.80) | 5.21(3.50) |
| SMM-AUD | -2.42(2.73) | -1.87(2.79) |
| SMM-CP | -0.29(3.29) | -0.45(3.02) |
| SMM-RT | 3.88(4.36) | 3.69(3.14) |
| SMM-SAL | 0.18(2.15) | 0.78(1.63) |
| SMM-DA | -0.02(2.67) | 1.01(1.91) |
| SMM-CO | -1.96(2.47) | -1.04(2.14) |
| VIS-FP | 2.66(2.24) | 2.93(2.15) |
| VIS-AUD | -1.57(2.70) | -1.11(2.07) |
| VIS-CP | -1.69(2.80) | -1.04(2.61) |
| VIS-RT | 1.58(2.62) | 0.89(2.40) |
| VIS-VA | -2.40(4.16) | -2.45(3.55) |
| VIS-SAL | -1.82(4.87) | -1.10(3.89) |
| VIS-DA | -1.21(2.46) | -0.86(2.78) |
| VIS-CO | 3.54(2.96) | 3.20(2.68) |
| FP-AUD | 3.13(3.92) | 2.57(4.16) |
| FP-CP | -2.20(3.93) | -2.18(4.43) |
| FP-RT | 1.90(2.82) | 1.80(2.12) |
| FP-VA | -1.94(2.16) | -1.53(2.50) |
| FP-SAL | 2.00(1.91) | 1.58(1.95) |
| FP-DA | 0.17(2.70) | -0.01(2.83) |
| FP-CO | 0.26(2.24) | 0.77(1.55) |
| AUD-CP | -1.35(2.08) | -0.91(2.34) |
| AUD-RT | 1.12(2.80) | 0.70(2.09) |
| AUD-SAL | -0.94(1.85) | -0.36(1.80) |
| AUD-DA | 0.54(2.42) | 0.60(2.61) |
| AUD-CO | -2.15(2.64) | -1.89(2.55) |
| CP-RT | 3.61(3.45) | 3.26(2.89) |
| CP-VA | -0.90(2.54) | -0.01(1.89) |
| CP-SAL | -0.25(1.85) | 0.20(1.61) |
| CP-DA | 5.66(3.16) | 4.84(2.76) |
| CP-CO | 0.33(2.90) | -0.31(2.53) |
| RT-VA | 1.10(1.75) | 1.34(1.56) |
| RT-SAL | 5.32(2.96) | 5.39(3.75) |
| RT-DA | 1.93(2.05) | 1.89(2.07) |
| RT-CO | 1.45(1.67) | 1.42(1.36) |
| VA-SAL | -0.30(1.99) | -0.38(1.44) |
| VA-DA | -0.26(1.82) | -0.40(1.61) |
| VA-CO | -0.45(1.41) | -0.11(1.10) |
| SAL-DA | -0.69(2.14) | -0.22(1.52) |
| SAL-CO | 3.18(2.02) | 2.35(1.78) |
| DA-CO | -0.40(1.57) | -0.46(1.86) |

**Bolded systems were significantly different between groups**

AUD=Auditory

CO=Cingular-Opercular

CP=Cingulo-Parietal

DA= Dorsal Attention

DM= Default Mode

FP=Frontoparietal

RT= Retrosplenial-Temporal

SAL=Salience

SMH= Somatomotor – Hand

SMM= Somatomotor – Mouth

VA= Ventral Attention

VIS=Visual
