# Supplementary material for: Globally weaker and topologically different: resting-state connectivity in youth with autism
Source: Mol Autism. 2017 Jul 26;8:39. doi: 10.1186/s13229-017-0156-6 (PMC5530457; doi:10.1186/s13229-017-0156-6)
Supplement: Supplementary file 5 — Follow-up groupdiff IQ matched. Means of cross-system functional connectivity for normalized correlations by group. (DOCX 65 kb) [file 13229_2017_156_MOESM5_ESM.docx]

Table S4. Follow-up analyses with matched subset matched within 12 months of age, 1 SD in IQ, and gender (when possible)

| Analysis | ASD  *M*(SD) | TDC  *M*(SD) | *F* | p-value* | η^2^_p_ | 95% CI |
| --- | --- | --- | --- | --- | --- | --- |
| Overall strength | 0.05 (0.02) | 0.07 (0.04) | 10.89 | 0.001 | 0.09 | -0.01, -0.03 |
| VA | 8.40 (4.17) | 5.89 (3.11) | 12.68 | 0.007 | 0.10 | 1.18, 3.84 |
| RT | 18.78 (8.68) | 14.48 (7.94) | 6.34 | 0.079 | 0.05 | 1.30, 7.32 |
| VA-SMM | 2.67 (2.11) | 1.66 (1.55) | 8.22 | 0.045 | 0.07 | 0.34, 1.68 |
| DM-Auditory | -1.41 (3.07) | -0.02 (2.14) | 7.58 | >0.10 | 0.06 | -2.35, -0.43 |
| VA-Auditory | -1.22 (2.43) | -0.20 (2.58) | 3.74 | >0.10 | 0.03 | -1.92, -0.11 |

CI=confidence interval; η^2^_p_=partial eta-square; PC=participation coefficient

*p-value for Overall strength is raw p-value, while p-values for individual systems are FDR corrected for multiple comparisions across all within-system comparisons (12) and cross-system comparisons (66).
