# Supplementary material for: Globally weaker and topologically different: resting-state connectivity in youth with autism
Source: Mol Autism. 2017 Jul 26;8:39. doi: 10.1186/s13229-017-0156-6 (PMC5530457; doi:10.1186/s13229-017-0156-6)
Supplement: Supplementary file 6 — Follow-up correl IQ matched. Means of cross-system functional connectivity for normalized correlations by group. (DOCX 41 kb) [file 13229_2017_156_MOESM6_ESM.docx]

Table S5. Follow-up non-parametric correlation analyses with ASD symptoms on ASD subset that was matched within 12 months of age, 1 SD in IQ, and gender (when possible)

| Functional System | ADOS Total (Raw) | *p*-value |
| --- | --- | --- |
| Overall strength | -0.33 | 0.010 |
| RT | 0.28 | 0.032 |
| VA | 0.27 | 0.038 |
| VA-SMM | 0.16 | 0.23 |
| DM-Auditory | -0.20 | 0.13 |
| VA-Auditory | -0.10 | 0.45 |
